# Increased testicular blood flow maintains oxygen delivery and avoids testicular hypoxia in response to reduced oxygen content in inspired air

**DOI:** 10.1038/s41598-018-29248-2

**Published:** 2018-07-19

**Authors:** G. Rizzoto, C. Hall, J. V. Tyberg, J. C. Thundathil, N. A. Caulkett, J. P. Kastelic

**Affiliations:** 1 Faculty of Veterinary Medicine, Department of Production Animal Health, University of Calgary, Calgary, AB Canada; 2 Departments of Cardiac Sciences and Physiology/Pharmacology, Libin Cardiovascular Institute of Alberta, University of Calgary, Calgary, AB Canada; 3 Faculty of Veterinary Medicine, Department of Veterinary Clinical and Diagnostic Sciences, University of Calgary, Calgary, AB Canada

## Abstract

Despite a long-standing assertion that mammalian testes operate near hypoxia and increased testicular temperature causes frank hypoxia, we have preliminary evidence that changes are due to hyperthermia *per se*. The objective was to determine how variations in inspired oxygen concentration affected testicular blood flow, oxygen delivery and extraction, testicular temperature and lactate production. Eight rams were maintained under general anesthesia, with successive decreases in oxygen concentration in inspired air (100, 21 and 13%, respectively). As oxygen concentration decreased from 100 to 13%, there were increases in testicular blood flow (9.6 ± 1.7 vs 12.9 ± 1.9 ml/min/100 g of testis, P < 0.05; mean ± SEM) and conductance (normalized flow; 0.46 ± 0.07 to 1.28 ± 0.19 ml/min/mm Hg/100 g testis (P < 0.05). Increased testicular blood flow maintained oxygen delivery and increased testicular temperature by ~1 °C; this increase was correlated to increased testicular blood flow (r = 0.35, P < 0.0001). Furthermore, oxygen utilization increased concomitantly and there were no significant differences among oxygen concentrations in blood pH, HCO_3_− or base excess, and no effects of venous-arterial differences in lactate production. In conclusion, under acute hypoxic conditions, testes maintained oxygen delivery and uptake by increasing blood flow and oxygen extraction, with no evidence of anaerobic metabolism. However, additional studies are needed to determine longer-term responses and potential evidence of anaerobic metabolism at the molecular level.

## Introduction

Maintenance of testicular temperature 3–4 °C lower than the body core temperature is essential for production of morphologically normal and motile sperm in most mammals^1–3^. There is a long-standing paradigm that the testicular microenvironment functions on the brink of hypoxia^4^ and that with increasing testicular temperature, there is increased testicular metabolism and increased oxygen demands, but no change in testicular blood flow. Thus, decreases in percentage of morphologically normal and motile sperm that follow testicular hyperthermia are usually attributed to secondary effects of hypoxia and not directly to hyperthermia^5,6^.

With ischemic conditions due to compromised blood flow caused by obstruction of testicular vessels (e.g., varicocele and testicular torsion) or hypobaric hypoxia (e.g., reduced oxygen pressure at high altitudes), spermatogenesis and fertility were impaired, similar to changes after testicular warming^7–10^. Notwithstanding, these observations were not clear evidence that the pathogenesis of increased testicular temperature was due to hypoxia.

Markers of hypoxia have been detected after exposure to hyperthermia^6,11^, supporting the assertion that effects of testicular hyperthermia are due to hypoxia. However, in those studies, neither testicular blood flow nor oxygen delivery/utilization were measured. Although hyperemia was reported when testes not covered by the scrotum were exposed to increased temperatures^12^, this was not regarded as sufficient evidence to challenge the classical view that hypoxia mediates damage caused by testicular hyperthermia. In a previous preliminary study^13^, conscious rams breathed inspired air containing 85, 21 or 14% oxygen for 30 h. Half of the rams had an insulated scrotum (a well-established model to increase testicular temperature); in those rams, percentages of morphologically normal sperm and motile sperm were significantly decreased from ~2 to 5 wk after exposure. Furthermore, in that study, hyperoxia did not mitigate effects of scrotal insulation, nor did hypoxia cause subsequent decreases in morphologically normal or motile sperm. Based on that preliminary study, we inferred that hyperthermia *per se*, and not hypoxia, was the underlying cause of reductions in morphologically normal and motile sperm following testicular hyperthermia. However, neither testicular blood flow nor oxygen delivery were measured. Therefore, objectives of the current study were to determine how variations in oxygen concentrations in inspired air affected testicular blood flow, oxygen delivery and extraction, lactate production and testicular temperature.

## Results

**Figure 1:**
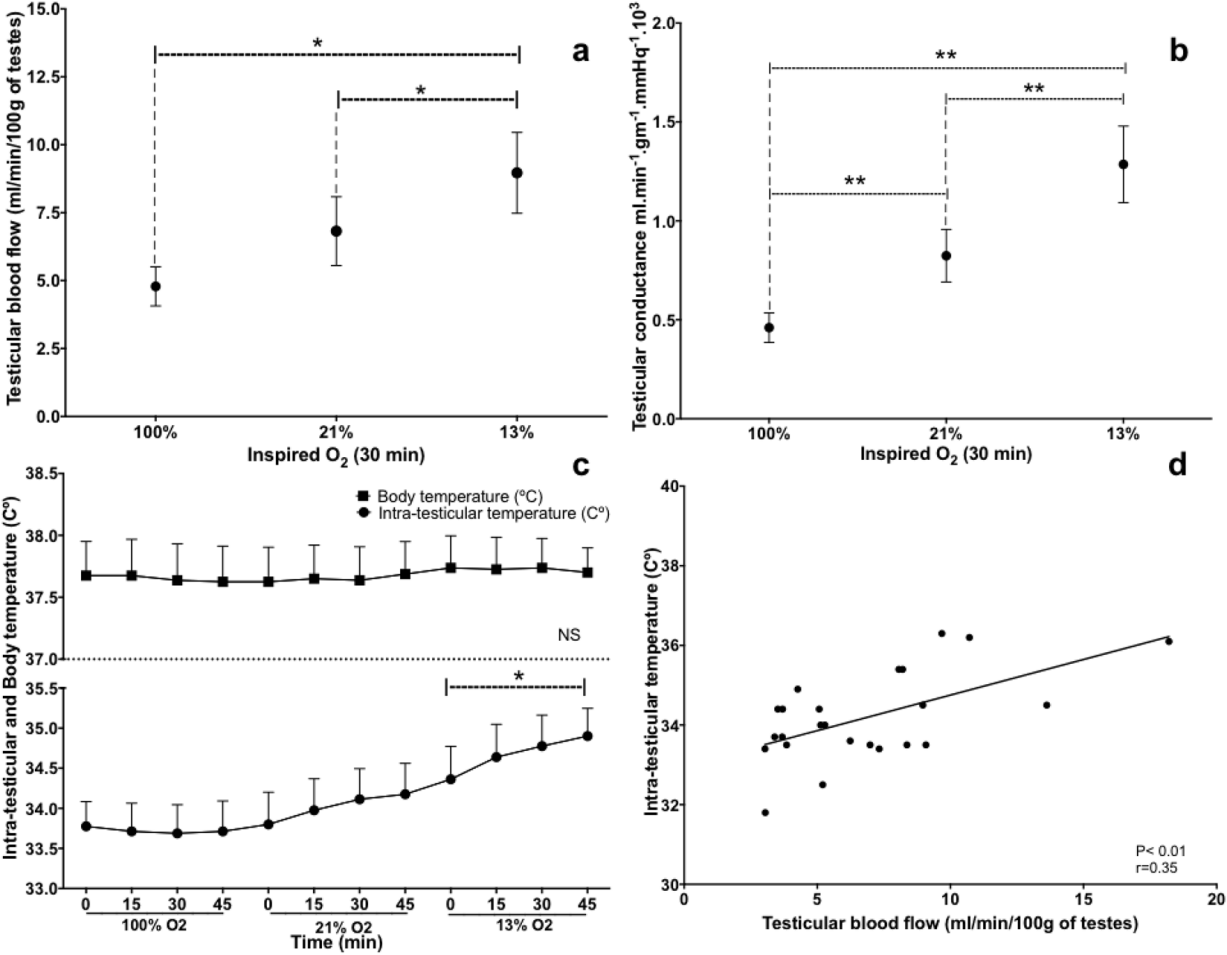
(**a**) Testicular blood flow and (**b**) testicular conductance (mean ± SEM) in eight rams exposed to three concentrations of oxygen in inspired air. (**c**) Testicular and body temperatures over time (mean ± SEM). (**d**) Correlation between testicular temperature and testicular blood flow (r = 0.35, P < 0.0001). One-way analysis of variance for repeated measures, followed by a Dunnet’s *t* test, was used to compare, among groups, data recorded at 30 min. Pearson’s correlation analyses were used to determine linear correlations. *P ≤ 0.05; **P ≤ 0.01.

As oxygen content in inspired air decreased, there were significant increases in testicular blood flow (Fig. 1a) and conductance (Fig. 1b), accompanied by concurrent increases in intra-testicular temperature (Fig. 1c), although body temperature remained constant. Furthermore, testicular blood flow and intra-testicular temperature were correlated (r = 0.35, P < 0.0001; Fig. 1d).

**Figure 2:**
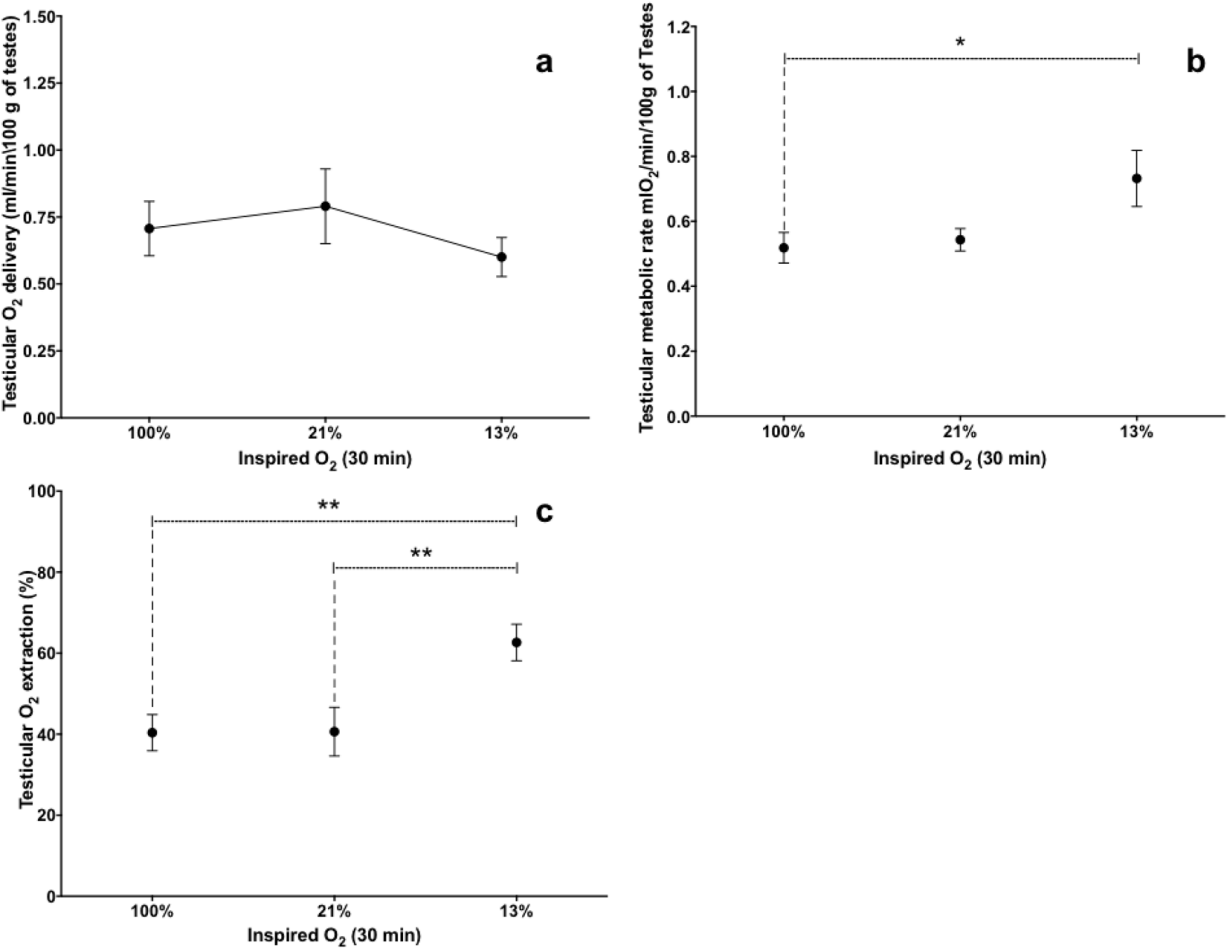
(**a**) Testicular oxygen delivery, (**b**) testicular metabolic rate and (**c**) testicular oxygen extraction (mean ± SEM) in eight rams exposed to three concentrations of oxygen in inspired air. One-way analysis of variance for repeated measures, followed by a Dunnet’s *t* test, was used to compare, among groups, data recorded at 30 min. *P ≤ 0.05; **P ≤ 0.01.

Testicular oxygen delivery was maintained throughout the entire experiment and not affected by reductions in oxygen concentration in inspired air (Fig. 2a). Furthermore, as oxygen concentrations decreased, there were increases in testicular metabolic rate (Fig. 2b) and oxygen extraction (Fig. 2c).

**Figure 3:**
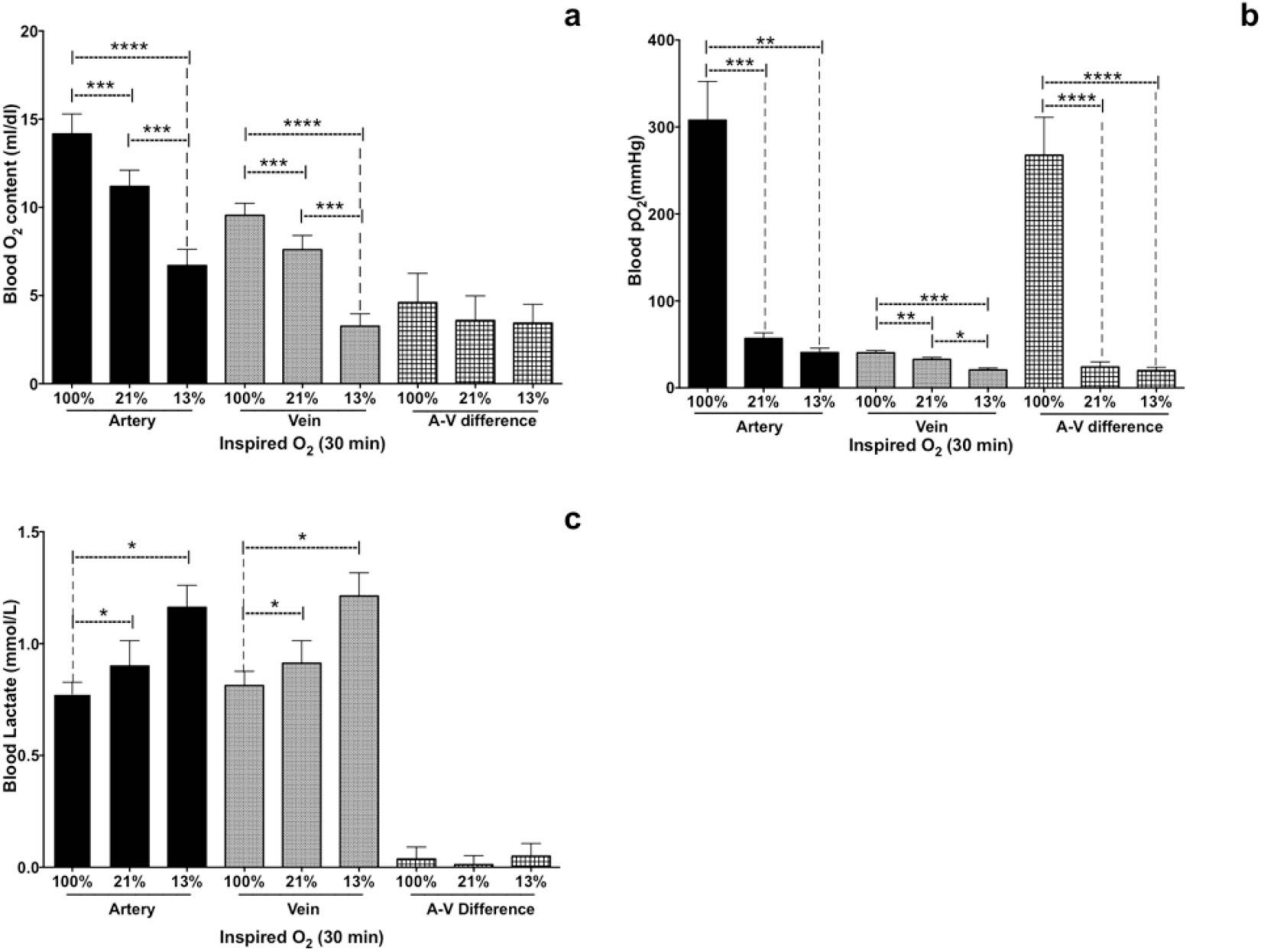
Testicular arterial, venous, and A-V difference in (**a**) venous blood oxygen content, (**b**) P_O2_; and (**c**) lactate concentrations and net production (mean ± SEM) in eight rams exposed to three concentrations of oxygen in inspired air. One-way analysis of variance for repeated measures, followed by a Dunnet’s *t* test, was used to compare, among the three groups, data recorded at 30 min. *P ≤ 0.05; **P ≤ 0.01; ***P ≤ 0.001; ****P ≤ 0.0001.

Consistent with our experimental design, there were large and significant differences among groups for testicular arterial and venous oxygen content (Fig. 3a) and arterial and venous P_O2_ (Fig. 3b). However, there were no significant differences among groups for P_CO2_ in either the testicular artery or vein.

Lactate concentrations in the testicular artery and the testicular vein were higher (P < 0.05) at 13% oxygen versus at 21 or 100%, but there was no significant difference among groups for venous-arterial differences in lactate concentrations (Fig. 3c). Furthermore, there were no significant differences among groups for arterial or venous pH, HCO_3_− or base excess, and no significant venous-arterial differences for pH, HCO_3_− or base excess.

## Discussion

Although regulation of blood flow in response to varying oxygen concentrations in inspired air has been extensively studied in several organs^3,14–17^, this was apparently the first study to characterize effects of varying inspired-air oxygen concentrations on testicular blood flow, oxygen delivery, oxygen uptake, and temperature in rams. In this study, as oxygen concentrations in inspired air decreased from 100 to 13%, blood flow increased concurrently and sustained oxygen delivery to the testis. This increased blood flow increased testicular temperature by ~1 °C and concomitantly increased metabolism and oxygen utilization. There was a small (albeit significant) increase in venous lactate concentrations, but no significant increase in the venous-arterial difference. Therefore, under the acute conditions of this study, we concluded that there were no indications of a shift to anaerobic metabolism over a broad range of inspired-air oxygen concentrations.

In the present study, hypoxia (i.e., 13% oxygen) significantly increased the supply of blood to the testes, either measured as blood flow or as conductance (flow normalized by arterial pressure). Although testicular hypoxia is considered to cause male infertility^18–20^, repeated complete interruptions of blood flow to the testes (occlusion of testicular artery for 1-h intervals) did not cause long-term impairment of sperm production in rams^21^. Regardless, chronic exposure to severe intermittent hypoxia in male rats (5% oxygen in inspired air) reduced sperm motility and fertility^22^. Perhaps the degree and duration of testicular hypoxia affects the ability to compensate.

Testicular blood flow and testicular temperature were significantly correlated (r = 0.35) in the current study, similar to bulls^23^. Clearly, hypoxia-induced increases in blood flow increased testicular temperature and metabolism. Furthermore, rectal temperature remained constant, suggesting that increased blood flow to the testis was not a systemic response, consistent with a previous report^23,24^. Acute or chronic hypoxia decreased oxygen content in rat muscle, although blood flow was not altered^25^. In the human brain, hypoxia (14% for 18 min) increased oxygen metabolic rate and blood flow (compared to 21% oxygen^26^, consistent with the present study. In some organs, a reduced oxygen supply increased blood flow^27,28^ due to release of vasodilators (decreased oxygen impairs re-phosphorylation of adenosine diphosphate (ADP), which is subsequently degraded to adenosine, a compound that causes vasodilation^15^.

Another consequence of induced hypoxia was increased oxygen extraction from arterial blood, from ~40% at 21% oxygen to ~60% at 13% oxygen (P < 0.01), thereby reducing oxygen content and P_O2_ in the testicular vein, although P_CO2_ remained unchanged. Observed effects were similar to those reported by Pittman^15^ and Hoffman^29^, indicating that under hypoxic conditions, the first physiological reflex was to open capillaries to increase available area for oxygen exchange, accounting for the observed increase in oxygen extraction. Interestingly, prolonged exposure to reduced oxygen concentrations (e.g., at high altitudes) may result in adaptation with no increase in extraction after long-term exposure, suggesting a more important role for increased extraction under acute exposures^30^.

Both hyperoxia and hypoxia can cause oxidative damage and lipid peroxidation^11^. Chronic exposure to hyperoxia reduced P_O2_, pH and increased P_CO2_ in old versus young rats, indicating that age affects tolerance to this condition^31,32^. Furthermore, hyperoxia increased the activity of radical oxygen species and may impair diaphragm contractility, potentially affecting body oxygenation^20^. Exposure to ~100% oxygen in inspired air decreased blood flow to the rat brain^26,33^, reduced (~8–30%) cardiac blood flow in humans^34^, and caused a 7% reduction in renal blood flow^35^, all of which were consistent with our finding that testicular blood flow was lowest at 100% oxygen.

Lactate is a well-defined marker of hypoxia^36–38^. In bulls, lactate concentrations in the rete testis were similar to those in the blood^39^, although for humans they can be slightly higher^38^. Lactate is produced in testes as a product of glucose metabolism by Sertoli cells^40–42^; increased concentrations were reported in pathological conditions in rats, including cryptorchidism^43^ and hypoxia^44^. Furthermore, a severe reduction in lactate was also associated with infertility^45^. In the present study, lactate concentrations in the testicular vein were increased (P < 0.05) when rams were exposed to 13% oxygen. However, venous-arterial differences were not significant among groups, nor were there significant venous-arterial differences in pH, HCO_3_− or base excess. Therefore, there was no evidence of reduced oxygen concentrations in inspired air causing anaerobic metabolism.

In the present study, only blood samples were collected and analyzed. Although retrieval of testicular biopsies was considered, it was expected that this would cause hemorrhage and inflammation that could invalidate the study. Furthermore, we did not sample tissues to detect angiogenesis, as these changes were not detected at 1 d after the onset of hypoxia in mice^46^, although they were detected at 5 d after the onset of hypoxia in rats^47^.

There are alternative approaches to detect a change from aerobic to anaerobic metabolism. Important examples are genes that contain the Hypoxia-Response Element (HRE) in their promoter region; hypoxia is associated with increased gene expression of those specific genes, including Hypoxia Induced Factor (HIF) I and II^48,49^. Furthermore, identification of thiobarbituric acid reactive substances (TBARS) and Reactive Species of oxygen (ROS) are important markers of tissue hypoxia and oxidative damage in the testes^50,51^. Unfortunately, in the present work, due to the experimental design and the potential for damage, testicular biopsies were not collected. Therefore, in future studies, recovery of testicular tissue and assessment of cellular and molecular evidence of tissue hypoxia should be done. In addition, more prolonged exposure to hypoxia in rams to determine long-term effects on testicular tissue, sperm and blood testosterone concentrations, are indicated.

Our experiment was designed to minimize the impact of anesthesia. Each ram was initially subjected to 100% oxygen, followed by two successive reductions in oxygen content in inspired air; therefore, each ram served as its own control. In addition, anesthetic depth was maintained at as constant a plane as possible throughout the experiment. Furthermore, adult animals were used, since the testicular vascular cone (TVC) undergoes development until puberty^52,53^ and is fundamental for thermoregulatory capabilities of the testes^3^. To our knowledge, there are no studies in pre-pubertal animals regarding testicular blood flow response to heat stress.

In conclusion, under acute hypoxic conditions (13% oxygen in inspired air), the testis maintained oxygen delivery and uptake by increasing blood flow and oxygen extraction, with no indications of a shift to anaerobic metabolism. Similarly, in our previous study in conscious rams, exposure of control rams (no scrotal insulation) to 85, 21 and 14% oxygen for 30 h had no significant effect on semen quality^13^. Thus, we concluded that the testis compensated for decreased oxygen concentrations in inspired air, although this needs additional confirmation.

## Materials and Methods

Eight crossbred rams (12–15 mo, 40–56 kg) were used. Rams were pre-medicated with 8 μg/kg dexmedetomidine (Dexdomitor (0.5 mg/ml, Zoetis, Parsippany-Troy Hills, NJ, USA) and 2 mg/kg of alfaloxone (Alfaxan 10 mg/ml, Jurox Pty Ltd, Rutherford, NSW Australia) administered IM. After approximately 15 min, anesthesia was induced with 2 mg/kg of alfaloxone administered IV. Thereafter, rams were intubated, anesthesia was maintained by inhalation of isoflurane (1.0–2.0%; Fresenius Kabi Animal Health, Richmond Hill, ON, Canada) and a constant volume ventilator (Harvard Apparatus – 12 breaths/min – 10 ml/kg stroke volume) was used throughout the study. To reduce the depth of anesthesia needed, epidural analgesia (0.07 mg/kg of xylazine (Rompun, 20 mg/kg, Bayer, Mississauga, ON, Canada) in ~4 mL of saline) and local anesthetic blocks (bupivacaine (Bupivacaine, 2.5 mg/ml, Hospira Inc., Lake Forest, IL, USA), ~2 ml/site SC) were performed at incision sites. All rams were maintained under general anesthesia throughout the procedure and euthanized (saturated potassium chloride given IV under deep anesthesia) at the end of the study. This study was reviewed and approved by the University of Calgary Health Sciences Animal Care Committee (AC16-0010) and all methods were conducted in accordance with the guidelines.

Each ram was exposed to three oxygen concentrations (100, 21 and 13% sequentially) in inspired air, by combining oxygen and nitrogen. Following reductions in oxygen concentration, rams were allowed ~20 min to adapt, and thereafter exposure was maintained for another 45 min, with measurements recorded at 30 min (temperature measured at 0, 15, 30 and 45 min). Oxygen concentrations were determined with an oxygen analyzer (MySign^®^O, Wilmar, MV, Germany).

All invasive procedures were performed under anesthesia. Testicular temperatures were measured by inserting a needle thermocouple (20-gauge × 2.5 cm^23^), through the scrotal skin (anterior aspect of testis) and into the testis. This thermocouple was inserted at the beginning of the intervention and remained *in situ* throughout the study. Standard ECG leads were attached (for cardiac monitoring). The right carotid artery was isolated and a 14-gauge polyvinyl catheter was placed for monitoring arterial pressure and determining arterial blood gases. The right jugular was isolated for intravenous administration of saline (5 ml/kg/h) and drug administration. An incision (~12 cm) was made between the right external inguinal ring and attachment of the scrotum to the body wall. The spermatic cord was identified and the testicular artery and vein isolated. A 20-gauge catheter was inserted in the left testicular vein (distal to the testis) for blood gas and lactate measurement. Blood samples were collected from the carotid artery and testicular vein to measure blood gases and lactate (Nova Biomedical, Stat Profile^®^ pHOx Ultra^®^, Waltham, MA, USA). An ultrasonic flow probe (2SB1551; Transonic^®^ Flowprobe, Ithaca, NY, USA) was placed around testicular artery to measure blood flow. Measurements were performed and recorded and arterial and venous blood samples collected at 30 min after the start of the monitored interval. Blood flow was obtained with specific software (Sonometrics Corp. System, London, ON, Canada) and data were further converted using custom software (CV Works, AccuDAQ Inc, Calgary, AB, Canada). Testicular perfusion and oxygenation were calculated as described (Table 1). In addition, for testicular vasculature, arterial-venous differences were calculated for oxygen content, P_O2_, P_CO2_, pH, HCO_3_− or base excess and venous-arterial differences were calculated for lactate.

**Table 1:** Formulas for testicular perfusion and oxygenation^54,55^.

Testicular O_2_ delivery (ml/min)	TDO_2_ = Q(t)*CaO_2_/100
Testicular metabolic rate (ml/min)	TVO_2_ = Q(t)*(CaO_2_ − CvO_2_)/100
Testicular O_2_ extraction (%)	O_2_ extraction = TVO_2_/TDO_2_
Testicular conductance (mL/min^−1^/g^−1^/mmHq^−1^)	Testicular conductance = (Q(t)/testicular weight)/aortic blood pressure

^*^Q(t) = testicular blood flow (ml/min); CaO_2_ = arterial O_2_ content (ml/dl).

CvO_2_ = venous O_2_ content (ml/dl).

One-way analysis of variance for repeated measures, followed by a Dunnet’s *t*-test, was used to compare, among the three groups, data recorded at 30 min. Pearson’s correlation analysis was used to determine linear correlations. All statistical analyses were performed with GraphPad Prism Version 6.0 (GraphPad Software Inc, La Jolla, CA, USA) and P < 0.05 was considered significant.

### Data availability

The datasets generated during and/or analyzed during the current study are available from the corresponding author on reasonable request.
